# Stress-Corrosion-Cracking Sensitivity of the Sub-Zones in X80 Steel Welded Joints at Different Potentials

**DOI:** 10.3390/ma17143481

**Published:** 2024-07-14

**Authors:** Ci Zhang, Yinsheng He, Wenyue Zheng

**Affiliations:** National Center for Materials Service Safety, University of Science and Technology Beijing, Beijing 100083, China; zhangxiaoci1208@163.com

**Keywords:** X80 steel, welded joint, SCC initiation mechanism, anodic dissolution, hydrogen embrittlement

## Abstract

X80 steel plays a pivotal role in the development of oil and gas pipelines; however, its welded joints, particularly the heat-affected zone (HAZ), are susceptible to stress corrosion cracking (SCC) due to their complex microstructures. This study investigates the SCC initiation mechanisms of X80 steel welded joints under practical pipeline conditions with varying levels of cathodic protection. The SCC behaviors were analyzed through electrochemical measurements, hydrogen permeation tests, and interrupted slow strain rate tensile tests (SSRTs) conducted in a near-neutral pH environment under different potential conditions (OCP, −1.1 V_SCE_, −1.2 V_SCE_). These behaviors were influenced by microstructure type, grain size, martensite/austenite (M/A) constituents, and dislocation density. The sub-zones of the weld exhibited differing SCC resistance, with the fine-grain (FG) HAZ, base metal (zone), welded metal (WM) zone, and coarse-grain (CG) HAZ in descending order. In particular, the presence of coarse grains, low dislocation density, and extensive M/A islands collectively increased corrosion susceptibility and SCC sensitivity in the CGHAZ compared to other sub-zones. The SCC initiation mechanisms of the sub-zones within the X80-steel welded joint were primarily anodic dissolution (AD) under open-circuit potential (OCP) condition, shifting to either hydrogen-enhanced local plasticity (HELP) or hydrogen embrittlement (HE) mechanisms at −1.1 V_SCE_ or −1.2 V_SCE_, respectively.

## 1. Introduction

To enhance the transportation efficiency of oil and gas pipelines and optimize construction costs, the use of high-strength steel with substantial wall thickness has emerged as a critical development focus. Notably, X80 steel has been successfully implemented in the West–East Gas Pipeline Project, thereby underscoring its paramount significance in terms of safety concerns. Pertinent data reveal that incidents involving pipe explosions and cracking frequently transpire at welded joints and adjacent areas along these pipelines [1,2]. The stress corrosion cracking of the welded joints of pipeline steel is an important cause of accidents. Gaining insights into the microstructure disparities within different sub-zones of the welded joint is helpful to elucidating the SCC failure mechanism and implementing preventive measures.

Previous investigations have examined the influence of microstructure type, grain size [3], dislocation density [4,5], M/A constituents [6,7], and other factors on SCC susceptibility. Simultaneously, evaluating SCC sensitivity requires considering the corrosion propensity and hydrogen diffusion properties of different sub-zones in the welded joint [8]. Lan et al. observed the change of the effective hydrogen diffusion coefficient under different heat inputs and considered that the coarsening of the structure would reduce the hydrogen embrittlement sensitivity of the material [9]. The findings of Jiang et al. demonstrated that the susceptibility to hydrogen-induced cracking in the heat-affected zone (HAZ) is attributed to its complex microstructure and higher hydrogen diffusion coefficient [8]. The extensive content and bulk state of M/A constituents in the CGHAZ act as stress concentrators and hydrogen traps, leading to greater SCC sensitivity [7,10]. Conversely, Azevedo et al. emphasized the microstructure sensitivity in crack propagation, observing more pronounced crack branching within the matrix compared to the HAZ [11]. Considering the complexity of the microstructure and its inconsistent influence on SCC performance, a comprehensive evaluation of these factors is necessary.

The SCC mechanism of pipeline steel in a near-neutral pH environment involves anodic dissolution and hydrogen embrittlement cracking [12,13]. Numerous studies support that the SCC initiation in pipeline steel within such an environment is primarily driven by anodic dissolution mechanisms [14,15], often enhanced by stress and hydrogen [16,17]. Tang et al. [17] employed micro-electrochemical experiments to verify that the anodic dissolution process of metal is promoted under hydrogen charging conditions. As the environmental hydrogen content increases, a transition from anodic dissolution to hydrogen embrittlement cracking as the dominant SCC mechanism may occur [18]. However, limited research has been conducted on the crack initiation stage specifically within the welded joint, thus necessitating further investigation.

This study investigates the corrosion and SCC behaviors of the welded joint of X80 steel in a near-neutral pH environment under OCP and cathodic protection conditions. Electrochemical tests, hydrogen permeation tests, and intermittent slow strain rate tensile tests (SSRTs) were conducted to analyze the corrosion properties, hydrogen diffusion coefficient, and SCC crack growth status of different sub-zones in the welded joint. Additionally, the mechanism of SCC crack initiation was analyzed by combining dislocation density with microstructure data obtained from X-ray diffraction (XRD), electron backscatter diffraction (EBSD), and scanning electron microscopy (SEM).

## 2. Materials and Methods

### 2.1. Materials and Solution

The pipeline steel utilized in this study is a low-carbon micro-alloyed X80 grade, with a yield strength exceeding 651 MPa, produced through the thermo-mechanical controlled process (TMCP). Welded joints were fabricated using the identical wire (H08MnMoTiB) and flux (JH-SJ101G/995N) in two passes at a welding speed of 1.3 cm/s. Its chemical composition is shown in Table 1.

The test solution used was NS4, simulating the electrolyte trapped under a disbanded coating of the SCC crack cluster in a pipeline site found in Canada. The chemical composition of the solution is as follows (g/L): KCl 0.122, NaHCO_3_ 0.483, CaCl_2_·2H_2_O 0.181, and MgSO_4_·7H_2_O 0.131. Before each experiment, the NS4 solution was deoxygenized for 1 h using high-purity nitrogen gas and then continuously exposed to a mixed gas consisting of 5% CO_2_ and 95% N_2_ to maintain the near-neutral pH environment.

### 2.2. Microhardness

Microhardness measurements were performed across the welded joint zone, spanning from the BM zone through the HAZ to the WM zone, using a Vickers hardness tester (HVD-10MP, Jujing, China) equipped with a pyramidal diamond indenter under a load charge of 200 g. The first hardness point in the tested matrix was taken as the reference point, and subsequent hardness values at different positions are obtained sequentially relative to this reference point. To ensure precision, each test was conducted in triplicate.

### 2.3. Intermittent SSRT

The intermittent SSRT was conducted on the Letry 20007-L25 test system machine (Letry, Xi’an, China) under various potentials and at room temperature. Detailed descriptions of the relevant operational procedures can be found in our previous work [19]. In the initial stage of the interrupted SSRT, a strain rate of 1 × 10^−6^/s was applied until the stress approached near the ultimate tensile strength (UTS) of the steel. Subsequently, during the second step of the experiment, the steel samples were exposed to a constant load nearly equivalent to UTS while being immersed in NS4 solution purged with a mixture of 5% CO_2_ and 95% N_2_ for 24 h.

**Figure 1:**
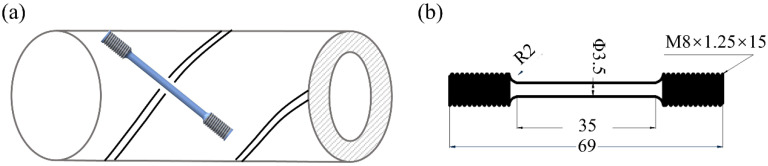
(**a**) Sampling position and (**b**) size for intermittent SSRT samples.

Sampling was conducted perpendicular to the girth weld of the X80 steel, with the longitudinal center of the sample positioned at the second-pass weld. Dog-bone tapered samples were machined for SSRTs, as illustrated in Figure 1a. The working section had a total length of 35.00 mm, and the gauge diameter measured 3.50 mm, as shown in Figure 1b. Before each test, tensile specimens underwent sequential grinding from coarse to fine grades, culminating in a final polishing stage using 1500-grit emery papers while keeping the polishing direction aligned parallel to the applied stress. To ensure precision, each condition was also tested at least three times.

### 2.4. The Electrochemical Test

The electrochemical test processes across the BM zone, FGHAZ, CGHAZ, and WM zone in the NS4 solution were investigated using a P4000 workstation (Gamry, Warminster, PA, USA). The OCP was measured for half an hour, followed by electrochemical impedance spectroscopy (EIS) tests over frequencies ranging from 10^5^ to 10^−2^ Hz with 10 mV amplitude. Potentiodynamic (PD) curves were obtained at a scanning speed of 0.33 mV/s from −0.6 V_SCE_ to 0.2 V_SCE_. The test surface, with an area of 1 cm^2^ cut from the BM zone, FGHAZ, CGHAZ, and WM zone, was used as the working electrode. Samples were polished to 1500 grit with sandpaper and cleaned with alcohol. A saturated calomel electrode (SCE) was used as the reference electrode, and a platinum sheet was used as the counter electrode. Welded joint sections were ground and polished and then etched with 4% nitric acid alcohol to reveal the microstructure. All electrochemical data were analyzed using Gamry Echem Analyst software version 6.33 (Warminster, PA, USA).

### 2.5. Hydrogen Permeation Test

**Figure 2:**
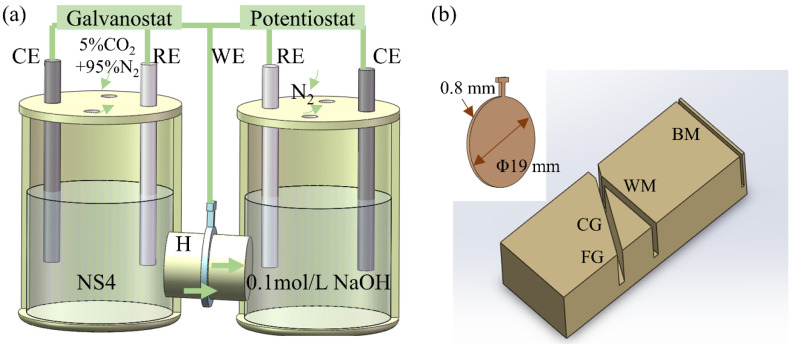
(**a**) Diagram of Devanathan-type double electrolytic cell for hydrogen permeation test, (**b**) sampling position and sample size.

The hydrogen permeation test was conducted using a Devanathan-type double electrolytic cell. A steel membrane, 0.80 mm thick and 19 mm in diameter, was inserted between two compartments of the cell, with each electrolyte exposed to an area of approximately 1.54 cm^2^ (1.4 cm in diameter), as shown in Figure 2a. The cathodic side of the cell contained NS4 solution purged with a gas mixture of 5% CO_2_ and 95% N_2_, while the anodic side of the cell was maintained at a constant potential of 0.3 V_SCE_ in 0.1 mol/L NaOH solution, bubbled with N_2_ to remove oxygen. Galvanostatic polarization at −1.1 V_SCE_ or −1.2 V_SCE_ was applied once the oxidation current density stabilized lower than 1 μA/cm^2^ to measure the hydrogen permeation flux. The resulting anodic current density provided a direct measurement of the hydrogen permeation flux.

From the hydrogen permeation curve, the parameters related to hydrogen diffusions, such as hydrogen permeability (J_∞_L), effective diffusion coefficient (D_eff_), and apparent solubility (C_0_) can be calculated by the following equations:

(1) $$J_{\infty }L=\frac{i_{\mathrm{ss}}\;L}{\mathrm{FA}}$$

(2) $$D_{\mathrm{eff}}=\frac{L^{2}}{6t_{\mathrm{lag}}}$$

(3) $$C_{0}=\frac{J_{\infty }L}{D_{\mathrm{eff}}}$$

where i_ss_ (μA/cm^2^) is the steady state hydrogen flux current density representing the maximum current flux that can be achieved, while L (mm) is the sample thickness, A (cm^2^) is the area of the specimen subjected to charging and oxidation, F (C/mol) is the Faraday constant, t_lag_ is the lag time taken for permeation current density to reach 0.63 times the steady-state value, and D_eff_ (cm^2^/s) is the lattice diffusion coefficient. In this paper, F = 96,500 C/mol was fixed [20].

The steel membranes, extracted from different sub-zones of the welded joint of X80 steel, were employed to investigate hydrogen permeation behaviors across various regions, including the BM zone, FGHAZ, CGHAZ, and WM zone, as illustrated in Figure 2b. Before conducting the hydrogen permeation test, the steel membranes underwent pre-treatment procedures involving grinding, cleaning on both sides, and nickel-plating on one surface only of each sample.

### 2.6. XRD Measurements

The dislocation density of the sub-zones of X80 steel welded joint was analyzed using micro-XRD (Smartlab, Rigaku, Japan). Before the experiment, electrolytic polishing was performed to release the residual strains introduced by mechanical polishing. During the XRD experiment, a probe with 500 μm grating slit was used, operating at a scanning voltage of 45 kV and current of 200 mA with a CuK_α1_ target. The scanning angle ranged from 20° to 120°, employing a scanning step of 1°/s and a scanning rate of 0.02°/s. The dislocation density can be calculated by using the full width at half maximum (FWHM) values in the XRD profile, which was expressed as:

(4) $$D=\frac{k\lambda }{\beta \cdot \cos\;\theta }$$

(5) $$\delta =\frac{1}{D^{2}}$$

where D, k, λ, β, and θ represent the crystal size, parameters, X-ray wavelength of the copper target, Bragg diffraction angle, and FWHM, and δ is the dislocation density. k is the constant of 0.94.

### 2.7. Microstructural Characterization

To characterize the various microstructures among the sub-zones within the X80 steel welded joints, all tested specimens were ground with 3000-grit paper and polished with 2.5 μm diamond suspension, degreased with alcohol, and etched with nital solution. The microstructures of the BM zone, FGHAZ, CGHAZ, and WM zone were examined using an optical microscope (OM, DM4000M, Leica, Germany). Additionally, the M/A constituent of the sub-zones within the X80 steel welded joint was characterized by SEM (SIGMA 300, ZEISS, Oberkochen, Germany) equipped with EBSD measurement system. The phase information of those sub-zones within the X80 steel welded joint was obtained by EBSD techniques at an accelerating voltage of 20 kV and a step size of 0.12 μm. The EBSD data were analyzed using CHANNEL 5 software (HKL Technology-Oxford Instruments, Abingdon, UK). To obtain a clear diffraction pattern by EBSD, the sample underwent mechanical polishing processing followed by vibration polishing with 50 nm SiO_2_ suspension. To investigate the crack initiation conditions in different sub-zones, the samples were cut along the rolling direction–transverse direction (RD-TD) plane and examined to observe the cross-sectional morphology of SCC cracks among the sub-zones after SSRT. The samples were mounted with conductive inlay and underwent similar sample preparation process as EBSD samples. The cross-sectional position and morphology of crack initiation were observed using SEM (SIGMA 300, ZEISS, Germany) equipped with a backscattering electron (BSE) detector.

## 3. Results

### 3.1. Microstructure of the Sub-Zones of X80 Steel Welded Joint

#### 3.1.1. Macroscopic Microstructure Analysis

**Figure 3:**
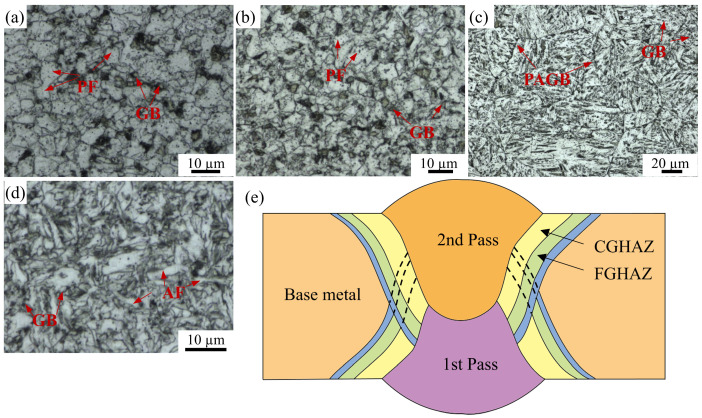
OM images of the sub-zones of X80 steel welded joint: (**a**) BM, (**b**) FGHAZ, (**c**) CGHAZ, (**d**) WM. (**e**) Schematic diagram illustrating the various sub-zones (B: bainite, GB: granular bainite, F: ferrite, AF: acicular ferrite, PF: polygonal ferrite, PAGB: prior austenite grain boundary).

The optical microscope images in Figure 3a–d show the sub-zones within the X80 steel welded joints, displaying various microstructures. Figure 3e presents a schematic diagram of the welded joint, indicating the formation of sub-zones following two welding processes. Near the second-pass welded area, the BM zone exhibits a microstructure comprising polygonal ferrite and granular bainite with an average grain size of 6.5 μm, as depicted in Figure 3a. Within the heat treatment zone, both the FGHAZ and CGHAZ are observed due to varying degrees of heat treatment influence. The FGHAZ, closer to the BM zone, undergoes a complete recrystallization during welding, resulting in uniform polygonal ferrite and granular bainite structures with an average size of 6 μm. The CGHAZ adjacent to the welding region (Figure 3c) exhibits clear prior austenite grain boundaries (PAGB) with an average size of 64 μm, containing granular bainite attributed to overheating during the second-pass weld process. The WM zone primarily consists of uniform acicular ferrite, supplemented with small-sized polygonal ferrite and granular bainite (Figure 3d).

#### 3.1.2. M/A Constituents

**Figure 4:**
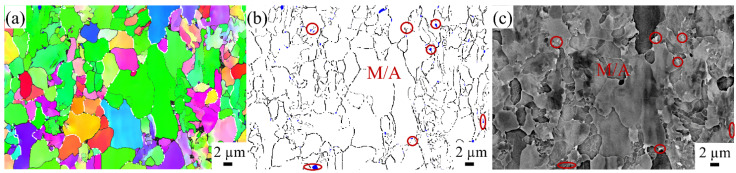
EBSD. (**a**) Inverse pole figure map, (**b**) phases distribution map, (**c**) backscattering electron map at the same position of BM zone of X80 steel welded joint.

The BM zone in X80 steel was analyzed using EBSD and BSE, as shown in Figure 4. The white line represents the prior austenite grain boundary, within which a fine polygonal ferrite sub-structure distribution is observed. The blue region in Figure 4b corresponds to the martensite–austenite (M/A) constituents with a face-centered cubic lattice. At the same time, the BSE diagram in Figure 4c shows the white island structure distributed along the grain boundary at the corresponding position and marked with red circles. Thus, the distribution of M/A constituents in different zones of X80 steel can be analyzed using BSE technology and a locally enlarged diagram.

**Figure 5:**
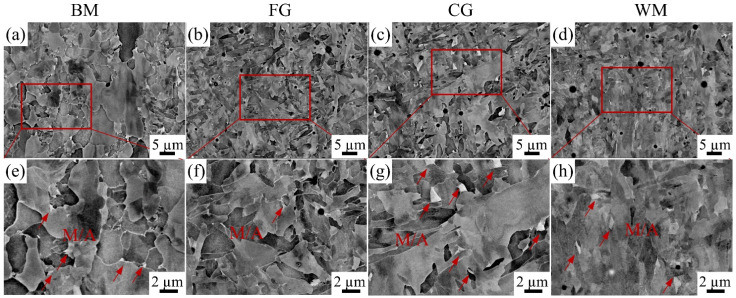
M/A constituents in different sub-zones of X80 steel welded joint: (**a**,**e**) BM, (**b**,**f**) FGHAZ, (**c**,**g**) CGHAZ, (**d**,**h**) WM.

Figure 5 shows the size, shape, and distribution of the second phases, namely M/A constituents in the BM zone, FGHAZ, CGHAZ, and WM zone. The morphology of the red box area in Figure 5a–d was locally enlarged, as shown in Figure 5e–h, and the M/A components were annotated with red arrows. It is evident that the FGHAZ exhibits a relatively lower abundance of M/A components compared to other zones. The number of M/A constituents in other zones is comparable. A previous study by Ramunni et al. [21] emphasized that not only the quantity but also the size distribution and morphology of inclusions significantly influenced the hydrogen diffusion behavior. The average size of M/A constituents in each zone is measured as 0.3 μm in the BM zone, 0.1 μm in the FGHAZ, 0.65 μm in the CGHAZ, 0.35 μm in the WM zone, respectively; notably, the CGHAZ exhibits a significantly larger average size compared to other regions. Moreover, it is noteworthy that both the BM zone and WM zone exhibit similar numbers and sizes of M/A constituents, which are comparatively smaller than those observed in the CGHAZ.

### 3.2. Vickers Microhardness of Sub-Zones within the X80 Steel Welded Joint

**Figure 6:**
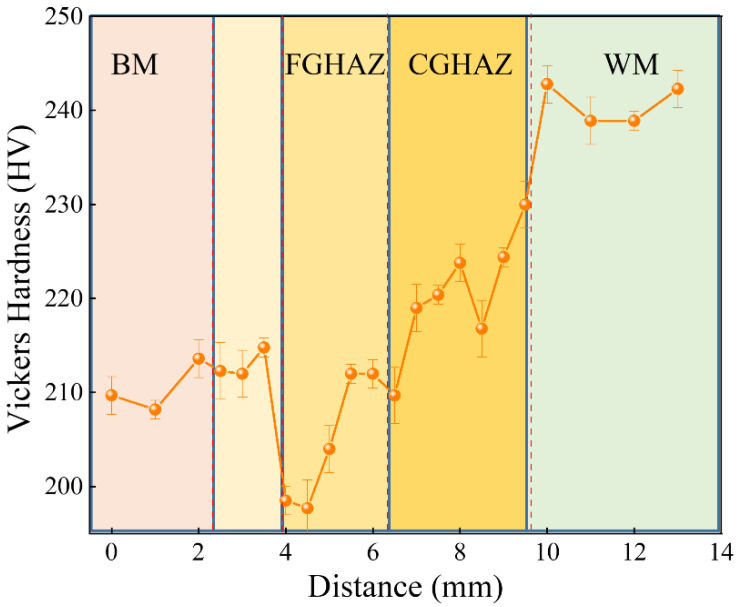
Microhardness of X80 steel welded joints.

Figure 6 illustrates the Vickers microhardness distribution of different sub-zones in the X80 steel welded joint. The WM zone shows a maximum hardness value of 242 HV due to its acicular ferrite structure, doped with a small amount of polygonal ferrite and granular bainite, formed during melting and solidification in thermal cycles. This hardness distribution aligns with the findings by Mohtadi [22]. Additionally, research by Park et al. [23] further demonstrates that homogeneous fine acicular ferrite with high dislocation density in the WM zone enhances its mechanical properties and hardness. Moving away from the WM zone, hardness decreases to a minimum of 197 HV in the FGHAZ. However, as we move even further away from the WM zone towards the BM position, there is an increase in hardness levels again, reaching approximately 210 HV.

### 3.3. SCC Behavior

**Figure 7:**
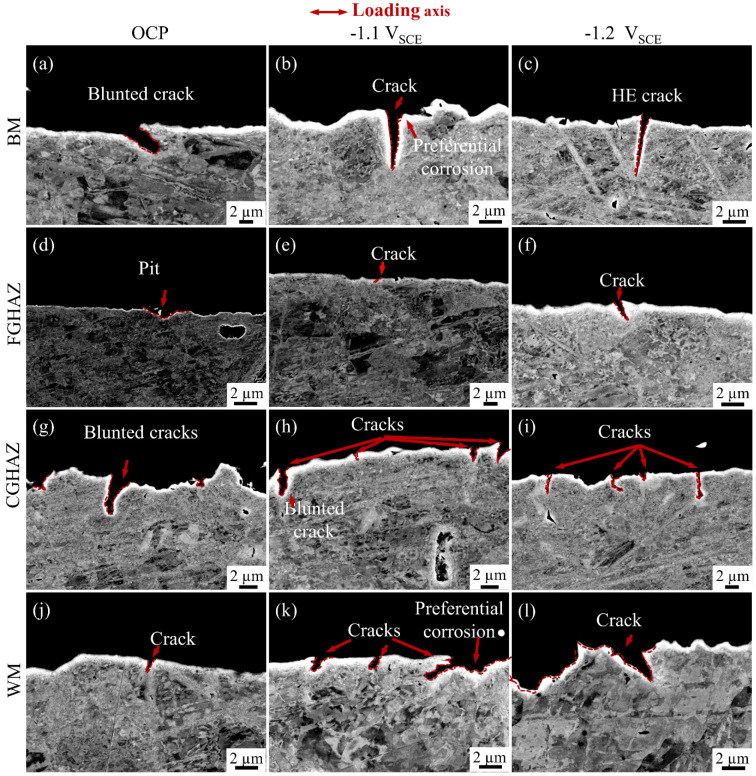
Cross-sectional BSE images of the sub-zones within the X80 steel welded joint showing typical crack morphology at OCP (**a**,**d**,**g**,**j**), −1.1 V_SCE_ (**b**,**e**,**h**,**k**), and −1.2 V_SCE_ (**c**,**f**,**i**,**l**) conditions. BM: (**a**–**c**); FGHAZ: (**d**–**f**); CGHAZ: (**g**–**i**); WM: (**j**–**l**).

The crack morphology of the sub-zones in the X80 steel welded joint was characterized under three different potential conditions: OCP, −1.1 V_SCE_, −1.2 V_SCE_, as depicted in Figure 7. Under OCP conditions (Figure 7a,d,g,j), clear corrosion characteristics were observed in each sub-zone, with the CGHAZ showing the most pronounced corrosion and cracking features. Cracks with signs of corrosion were also observed in both the BM and WM zones, while only pits were observed in the FGHAZ.

When the potential was shifted to −1.1 V_SCE_, the corrosion characteristics decreased across all sub-zones (Figure 7b,e,h,k). However, the CGHAZ still exhibits a higher number of cracks, with a maximum crack size of 3.0 μm and sharper crack tips compared to the OCP conditions. The FGHAZ shows minimal damage, with only some small notches observed. Cracks were observed in the WM zone, but their number and depth were lower than those in the CGHAZ. In the BM zone, although fewer in number, cracks reach a depth of 4.6 μm.

At −1.2 V_SCE_, the corrosion characteristics further diminished, while the depth and sharpness of cracks increased (Figure 7c,f,i,l). The hydrogen embrittlement (HE) characteristic was prominently observed in the cracks with no change in their number but an increased depth compared to that at −1.1 V_SCE_ condition. The CGHAZ continued to show the largest number of cracks, with a maximum crack depth of 4 μm, while other zones display fewer cracks but exhibit clear signs of hydrogen embrittlement (HE) characteristics.

During the entire test period (3.49 days), the SCC crack experienced incubation and initiation. Based on the crack depth of different zones under various hydrogen charging environment, it can be roughly obtained that at OCP, −1.1 V_SCE_ and −1.2 V_SCE_, the maximum crack growth rates of the BM zone are 1.08, 1.32, and 1.40 μm/h, respectively; for the FGHAZ, they are 0, 0, 0.29 μm/d, respectively; for the CGHAZ, they are 1.00, 0.81, 1.15 μm/d, respectively; and for the WM zone, they are 0.28, 0.57, 0.77 μm/d. It can be observed that the CG-HAZ has a larger crack density, while the BM zone shows a larger crack growth rate.

**Figure 8:**
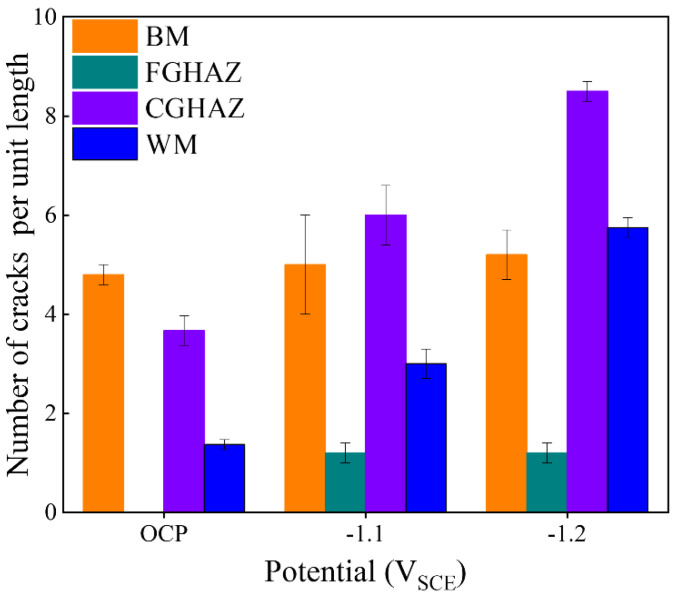
Number of cracks per unit length of sub-zones (BM, FGHAZ, CGHAZ, WM) in X80 steel welded joint at different potentials.

The crack density per unit length in the BM zone, FGHAZ, CGHAZ, and WM zone reflects the SCC sensitivity of different sub-zones within the welded joint under OCP, −1.1 V_SCE_, and −1.2 V_SCE_ conditions, as illustrated in Figure 8. Each statistical result was obtained from three replicates for accuracy. Under the OCP condition, the BM zone exhibits the highest number of cracks. However, there is no significant increase in crack formation under electrochemical hydrogen charging. The FGHAZ consistently displays the lowest crack density across all potential conditions, with no cracks observed under the OCP condition. The negative shift of the potential led to an increased occurrence of cracks in both the CGHAZ and the WM zones, with the CGHAZ being most significantly influenced, exhibiting a higher number of cracks. Based on the observed crack number in each sub-zone under various potential conditions, it can be concluded that the CGHAZ is more susceptible to SCC compared to other zones within the X80 steel welded joint.

### 3.4. Electrochemical Behavior

**Figure 9:**
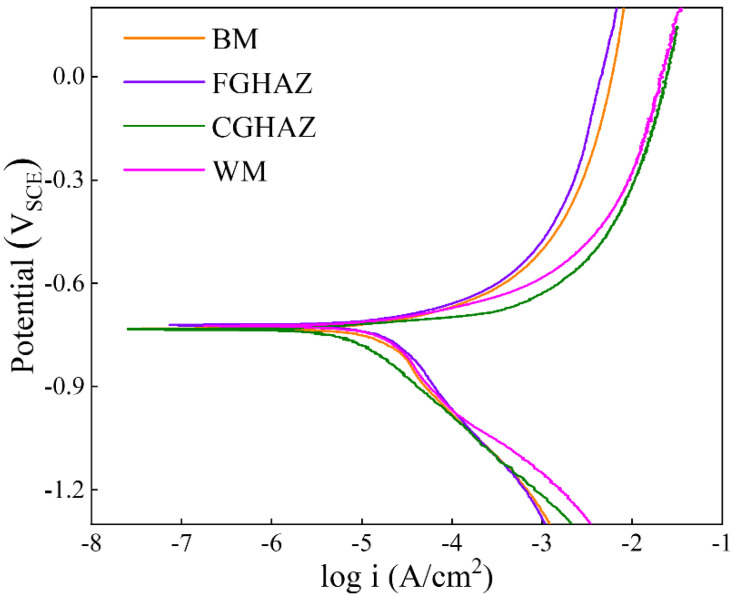
Polarization curves of the sub-zones within the X80 steel welded joint in NS4 solutions.

**Figure 10:**
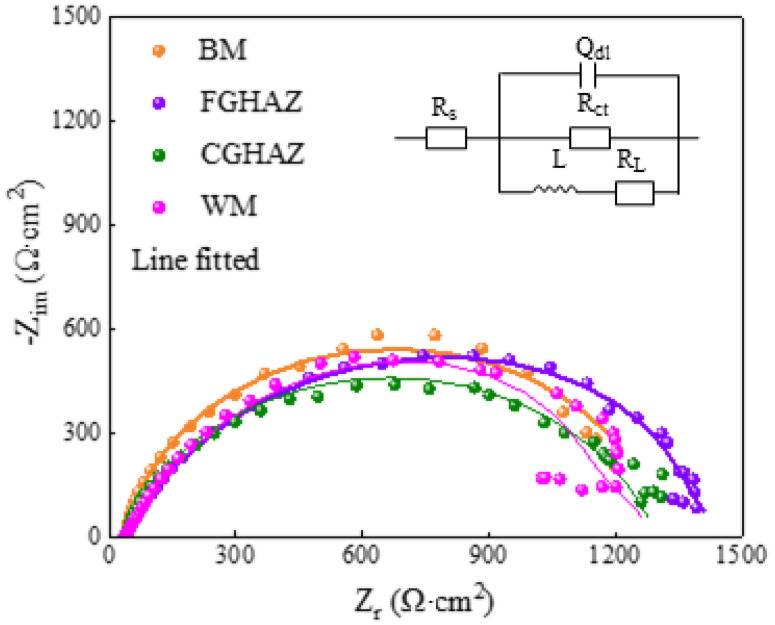
Nyquist diagrams of the sub-zones within the X80 steel welded joint in NS4 solutions and the equivalent circuit used for experimental result fitting.

The corrosion behavior of the sub-zones within the X80 steel welded joints in NS4 solutions was investigated through Potentiodynamic and EIS tests. As shown in Figure 9 and Table 2, the Potentiodynamic curves exhibit similar shapes, with the highest I_corr_ observed in the CGHAZ (15.37 μA), followed by the WM zone (13.55 μA), BM zone (11.87 μA), and FGHAZ (7.73 μA). Consequently, the FGHAZ demonstrates superior corrosion resistance compared to other sub-zones, while the CGHAZ exhibits the poorest corrosion resistance, consistent with the EIS results. The Nyquist diagrams measured on the sub-zones in the X80 steel welded joint in NS4 solution, along with the corresponding equivalent circuit used for fitting the experimental results, are presented in Figure 10. Here, R_s_ represents the solution resistance, CPE_dl_ denotes the double-layer capacitance, R_ct_ signifies the charge transfer resistance, and R_L_ and L represent the resistance and inductance associated with the adsorption process. The electrochemical parameters obtained from analyzing these Nyquist diagrams are listed in Table 3. The similarity in the impedance spectra across all regions indicates no significant variation in the corrosion products formed by different microstructures of the welded joint. The presence of an inductive arc can be attributed to the adsorption of corrosion products. Our findings reveal that CGHAZ exhibits the lowest charge transfer resistance, implying a higher susceptibility to corrosion.

### 3.5. Hydrogen Permeation Behavior

**Figure 11:**
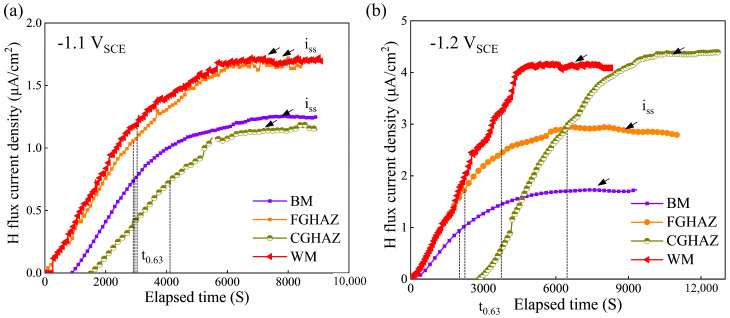
Hydrogen permeation current curve of BM, FGHAZ, CGHAZ, and WM zones of X80 steel welded joint under different potentials: (**a**) −1.1 V_SCE_, (**b**) −1.2 V_SCE_.

**Figure 12:**
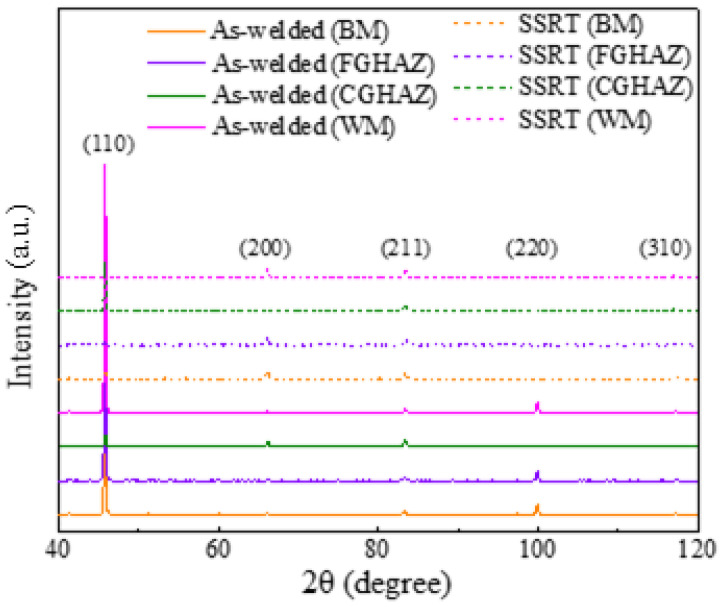
XRD peak profiles for different sub-zones in welded joints of X80 steel before and after SSRT.

The hydrogen permeation curves of the sub-zones in the X80 steel welded joint under −1.1 V_SCE_ and −1.2 V_SCE_ are illustrated in Figure 11. Based on those curves in Figure 11 and Equations (1)–(3), the calculated kinetic parameters for hydrogen permeation are listed in Table 4. The parameters i_ss_ and t_lag_ can be directly derived from the hydrogen permeation curve, as indicated in Figure 12. The penetration time i_ss_ is determined by extrapolating the linear portion of the transient permeation current rising curve to the cut-off point of the time coordinate axis, while t_lag_ represents the lag time required for permeation current density to reach 0.63 times the steady-state value. Furthermore, the parameters D_eff_ and C_0_ can be calculated by Equations (2) and (3). These parameters were compared to better understand the role of hydrogen in the SCC process of materials. With a negative shift in cathode potential, an increase in the stable current density (i_ss_) is observed in each zone. Under both −1.1 V_SCE_ and −1.2 V_SCE_ potentials, the CGHAZ exhibits the highest diffusion coefficient (D_eff_) and lowest hydrogen concentration (C_0_), while the FGHAZ shows the opposite trend. Considering the SCC susceptibility illustrated in Figure 7, it can be inferred that specimens with larger D_eff_ values had higher SCC susceptibility.

### 3.6. Analysis of Dislocation in Each Sub-Zone of X80 Steel Welded Joint

**Figure 13:**
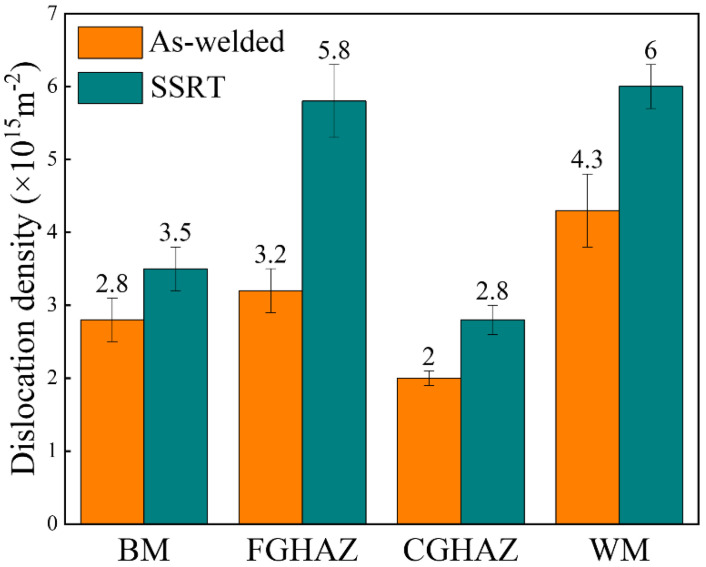
Histogram of the dislocation density of the sub-zones within the X80 steel welded joint.

The XRD profiles for different sub-zones within the X80 steel welded joints before and after SSRT show similar peaks at the same diffraction angles, as depicted in Figure 12. Despite their distinct microstructures, these sub-zones predominantly consist of main ferrite phase (body-centered cubic (BCC) structure) with a minor presence of M/A constituents (face-centered cubic (FCC) structure). Additionally, dislocations are crucial defects that significantly influence the service performance of materials, necessitating an analysis of their distribution in each sub-zone. The variations among the different sub-zones within the X80 steel welded joints before and after SSRT were visualized and illustrated through the calculation of the average dislocation density, as shown in Figure 13. Regardless of the SSRT process, the CGHAZ consistently displayed the lowest dislocation density (As-welded: 2.0 × 10^15^ m^−2^, SSRT: 2.8 × 10^15^ m^−2^), while the WM had the highest value (As-welded: 4.3 × 10^15^ m^−2^, SSRT: 6.0 × 10^15^ m^−2^). The dislocation density in each sub-zone increased following the SSRT process, which can be attributed to plastic deformation. This increase in dislocation density further facilitated a rise in hydrogen trap density, significantly influencing hydrogen permeation behavior within each sub-zone.

## 4. Discussion

### 4.1. Effect of Microstructure on Corrosion Properties of Sub-Zones within the X80 Steel Welded Joint

According to the results presented in Figure 7, the sub-zones exhibit prominent corrosion characteristics in terms of crack formation, indicating that anodic dissolution plays a pivotal role during the SCC initiation stage. Based on the electrochemical results of corrosion potential, polarization current density, and charge transfer resistance, the order of corrosion resistance is as follows: FGHAZ > BM > WM > CGHAZ. This finding aligns closely with previous studies conducted by Zhao et al. [3] and Wang et al. [24], which indicated that the corrosion process is significantly influenced by the type of microstructure, grain size, M/A composition, and dislocation density [25]. In the CGHAZ, overheating treatment leads to the presence of massive PAGB with granular bainite and M/A constituents, as shown in Figure 3c and Figure 5c,g. Coarse grain size and larger M/A constituents contribute to diminished corrosion resistance [10,26]. Furthermore, elevated internal residual stress can compromise the stability of surface corrosion product films, consequently accelerating corrosion [3]. Electrochemical characterizations of the charge-transfer resistance and corrosion potential have revealed that the corrosion tendency of the WM zone is second only to that of the CGHAZ, likely due to the presence of high dislocation density in the WM zone. Zhang et al. [27] analyzed the electrochemical corrosion tendency of different phase structures and concluded that ferrite exhibits a lower corrosion tendency compared to bainite. The microstructure in the BM zone primarily consists of polygonal ferrite with some smaller granular bainite, which exhibits corrosion characteristic cracking under the OCP condition, while the FGHAZ, which has undergone tempering heat treatment, is predominantly composed of polygonal ferrite and therefore only demonstrates minimal localized corrosion.

### 4.2. Effect of Microstructure on Hydrogen Permeation of Sub-Zones of the X80 Steel Welded Joint

The SCC susceptivity increases in various sub-zones within the welded joints of X80 steel, exhibiting a higher number of cracks or deeper crack depths and sharper crack tips, accompanied by a negative shift in cathodic potential, as illustrated in Figure 7. These findings highlight the crucial role of hydrogen in promoting the SCC process of X80 steel welded joint. Therefore, it is imperative to elucidate the hydrogen sensitivity of different sub-zones for a comprehensive understanding of the SCC mechanism. Evaluating local hydrogen embrittlement sensitivity necessitates an examination of hydrogen permeation behavior [28,29,30].

According to the result presented in Figure 11 and Table 4, the CGHAZ exhibits the most pronounced hydrogen diffusion coefficient at potentials of −1.1 V_SCE_ and −1.2 V_SCE_ compared to other sub-zones, while the hydrogen concentration stays at a lower level. The CGHAZ displays a distinctive coarse-grain structure (Figure 3) with fewer grain boundaries, along with coarse M/A constituents (Figure 13). The straight nature of grain boundaries within the CGHAZ facilitates a more direct path for hydrogen diffusion within the material. The presence of M/A constituents and defects within the material tend to facilitate trapping and aggregation of hydrogen atoms due to stress concentration at interfaces with the matrix, thereby increasing the risk of hydrogen-induced failure [31]. Furthermore, the susceptibility to hydrogen embrittlement is significantly influenced by the content of M/A constituents within the welded joint. The study conducted by Han et al. [29] demonstrates that a higher content of M/A constituents leads to a more dispersed distribution and lower D_eff_ value. Simultaneously, the limited quantity of these constituents restricts their ability to capture a significant number of hydrogen atoms, thereby limiting the achievable total hydrogen concentration. Additionally, it is widely acknowledged that dislocations, which are a prevalent defect in materials, are frequently employed as reversible hydrogen traps due to their low binding energy [32]. However, it has been observed that the CGHAZ exhibits relatively minor dislocation content compared to other sub-zones, which limits its ability to effectively capture sufficient amounts of hydrogen. Nevertheless, the higher hydrogen diffusivity in the CGHAZ indicates a faster hydrogen accumulation rate towards the crack tip [29].

The WM zone primarily consists of acicular ferrite, which exhibits a higher dislocation density compared to other phases and can effectively reduce internal hydrogen partial pressure through enhanced hydrogen dissolution. In the study of Wang et al. [33], it was also pointed out that the existence of acicular ferrite and fine M/A constituents in the WM zone resulted in greater hydrogen solubility than that in the HAZ and BM zone. Moreover, the grain boundaries within the acicular ferrite act as effective hydrogen traps and facilitate hydrogen diffusion, thereby maintaining a high solubility and diffusion coefficient for hydrogen. The FGHAZ exhibits a slightly lower dislocation density and smaller grain boundary compared to the BM zone, owing to its thorough heat treatment. Both the BM zone and FGHAZ possess similar microstructures characterized by polyferrite and granular bainite, with some M/A constituents incorporated. However, the FGHAZ displays a finer grain size but fewer M/A constituents. The two zones exhibit comparable hydrogen diffusion behavior that is influenced by their heterogeneous microstructure.

### 4.3. The SCC Behavior and Mechanism of the Sub-Zones within the X80 Steel Welded Joint

Some insensitive microstructures cannot crack even in a high-hydrogen-content environment, while some sensitive microstructures can crack in a low-hydrogen-content environment [22]. To comprehend the SCC mechanism of the X80 steel welded joints, a comprehensive characterization and analysis of crack growth morphology was conducted for each sub-zone under varying potentials (Figure 7). Influential factors, including dislocations, grain boundaries, grain size, and M/A islands, collectively exert a synergistic impact on the SCC process of the X80 steel welded joint. The coarse-grained microstructure in the CGHAZ under the OCP condition, characterized by a low dislocation density and the presence of prominent M/A islands, contributes to its susceptibility to SCC development, consistent with the findings reported by Zhao et al. [29] and Song et al. [34].

According to field investigation, SCC cracking of pipelines commonly occurs in the form of crack clusters. Small cracks gradually propagate and coalesce into larger ones, ultimately leading to failure. The initiation of cracks in the CGHAZ demonstrates a significantly heightened propensity for cracking compared to other sub-zones. The cracking tendency of materials is influenced by the corrosion and hydrogen sensitivity exhibited in different sub-zones [17,35]. The CGHAZ, characterized by a lower corrosion potential compared to other zones, experiences significant local dissolution under the OCP condition. Based on previous discussions, it can be inferred that the defect position within the CGHAZ is prone to the accumulation of hydrogen atoms, thereby increasing its susceptibility level to hydrogen-induced cracking. Upon application of the cathodic potential, the number of cracks gradually increases while retaining their corrosion characteristics. These observations collectively suggest that hydrogen facilitates anodic dissolution during SCC. The M/A constitute has greater SCC sensitivity due to its higher hardness and element enrichment [36]. The stress/strain concentration will be induced at the interface between M/A component with high hardness and soft matrix under the action of external tensile force [37,38].

The presence of a higher proportion of granular bainite in the WM zone results in a higher hardness level [22]. The WM zone exhibits a corrosion tendency second only to the CGHAZ, with a preferential occurrence of corrosion within the grains. Due to the presence of acicular ferrite in the WM zone, it exhibits smaller crack depth and good crack propagation resistance. The BM zone exhibits less local dissolution and fewer cracks; however, the depth of individual cracks is greater than that in other zones. There are more polygonal ferrite phases in the BM zone, which exhibit lower hardness and may result in a shorter crack incubation period and a larger crack depth. The hydrogen dissolved in the BM zone accumulates at stress concentration sites within the corrosion pit, thereby promoting crack propagation through accelerated anodic dissolution. The FGHAZ zone at OCP exhibited solely local corrosion dissolution without any evidence of cracking, indicating a low susceptibility to SCC. Conversely, other zones displayed the development of crack morphology after initial corrosion dissolution, highlighting the pivotal role played by corrosion in the initiation stage of crack formation. Several minute crack-like features emerged in the FGHAZ region at −1.1 V_SCE_ due to an increased presence of hydrogen in the surrounding environment. Despite a higher concentration of hydrogen at the −1.1 V_SCE_ potential, it fails to completely impede the process of anodic dissolution. Even under the condition of −1.2 V_SCE_, only a small number of tiny cracks appear at the FGHAZ, indicating the minimum SCC sensitivity.

In summary, the SCC process in X80 steel welded joints is influenced by different mechanisms under various potential conditions. At OCP, the process is controlled by anodic dissolution. When the potential shifts negatively to −1.1 V_SCE_, hydrogen assists in the initiation of SCC microcracks through the hydrogen-enhanced local plasticity (HELP) mechanism alongside anodic dissolution [6]. At −1.2 V_SCE_, hydrogen embrittlement will become the dominant mechanism of crack initiation.

## 5. Conclusions

The SCC sensitivity of the X80 steel welded joints was studied using various testing and characterization methods. The conclusions are as follows:

1. The sub-zones within the X80 steel welded joint exhibited varying degrees of SCC resistance, with crack density ranked in descending order as follows: fine-grain heat-affected zone, base metal zone, welded metal zone, and coarse-grain heat-affected zone. Additionally, it was observed that the base metal zone showed maximum SCC sensitivity based on the crack depth analysis.

2. The coarse-grain heat-affected zone of X80 steel welded joints exhibits a higher propensity for corrosion and increased sensitivity to stress corrosion cracking, attributed to the combination of coarse grains, low dislocation density, coarse M/A islands, and other defects.

3. The SCC initiation mechanisms in the sub-zones within X80 steel welded joints are dominated by anodic dissolution under the OCP condition. As the potential shifts to −1.1 V_SCE_ or −1.2 V_SCE_, the mechanisms transition to either hydrogen-enhanced local plasticity or hydrogen embrittlement, respectively.

## Figures and Tables

**Table 1:** Chemical composition of X80 steel welded joint (wt. %).

Material	Chemical Composition (wt. %)											
	C	Mn	Si	P	S	Mo	Ni	Cr	Cu	Nb	Al	Fe
X80	0.078	1.60	0.30	0.009	0.007	0.11	0.084	0.18	0.15	0.025	0.024	bal.

**Table 2:** Electrochemical parameters fitted from the polarization curves.

Zone	E_corr_ (mV_SCE_)	I_corr_ (uA/cm^2^)	Beta A (mV_SCE_/dec)	Beta C (mV_SCE_/dec)
BM	−739.25	11.87	74.28	175.05
FGHAZ	−733.80	7.73	60.16	177.424
CGHAZ	−746.12	15.37	80.03	155.44
WM	−745.15	13.55	86.40	175.53

**Table 3:** Electrochemical parameters fitted from the Nyquist diagrams.

Zone	R_s_ (Ω·cm^2^)	CPE_dl_ (μF·cm^2^·S^n−1^)	n	R_ct_ (Ω·cm^2^)	R_L_ (Ω·cm^2^)	L (H·cm^2^)
BM	37.18	0.00037	0.750	1576	2741	1.374 × 10^4^
FGHAZ	36.09	0.00034	0.739	1865	2035	389.9
CGHAZ	38.26	0.00030	0.827	1237	6258	0.01074
WM	37.67	0.00043	0.897	1275	1242	0.01177

**Table 4:** Results from permeation experiment for BM, FGHAZ, CGHAZ, and WM zones of X80 steel welded joint under −1.1 V_SCE_ and −1.2 V_SCE_ potentials.

Potential (V_SCE_)	Zone	i_ss_ (μA/cm^2^)	t_lag_ (s)	D_eff_ (×10^−6^ cm^2^/s)	C_0_ (ppm)
−1.1	BM	1.24	3062	3.38	0.14
	FGHAZ	1.66	3020	3.36	0.52
	CGHAZ	1.13	4089	9.10	0.145
	WM	2.65	3110	5.4	0.36
−1.2	BM	1.86	2130	3.38	0.63
	FGHAZ	2.85	2310	2.50	1.13
	CGHAZ	4.33	6451	6.48	0.58
	WM	3.11	4020	4.40	0.71

## Data Availability

Data are available on request to the corresponding author.
